# Virtual reality cognitive & functional assessment in psychosis

**DOI:** 10.1192/bjo.2021.769

**Published:** 2021-06-18

**Authors:** Sukhwinder Shergill, Lilla Porffy, Gabriella Whomersley, Timea Szentgyorgyi, Elias Mouchlianitis, Joel Patchitt

**Affiliations:** Institute of Psychiatry, Psychology and Neuroscience, King's College London

## Abstract

**Aims:**

To compare the MATRICS Consensus Cognitive Battery (MCCB) and a novel Virtual Reality (VR) task, called VStore, in assessing cognition and functional capacity (FC) in schizophrenia. We hypothesise that VStore reliably discriminates between patients and controls, correlates with the MCCB, and is well-tolerated. Additionally, VStore is expected to strongly correlate with FC measures.

**Background:**

Cognitive and functional deficits in schizophrenia have a major impact on everyday functioning of patients. The gold-standard cognitive assessment is the MCCB, while the USCD Performance-Based Skills Assessment (UPSA) is used to assess FC in this patient group. Neither of which are without limitations. For example, both take a long time to administer, and the MCCB alone cannot give clear indications of FC. We propose the use of a novel VR task to simultaneously measure cognition and FC in a single assessment. VStore is a shopping task, which involves a verbal learning task followed by buying items from a predetermined shopping list in a virtual minimarket.

**Method:**

Ten patients with schizophrenia or schizoaffective disorder and ten age/gender-matched healthy controls recruited from South London, completed the following assessments: VStore, MCCB, UPSA & Global Assessment of Functioning (GAF), and VR-Symptom Questionnaire (VRSQ); while controls only completed the VR task. To test whether VStore can differentiate between patients and controls we employed unpaired t-test. To explore associations between VStore Total Time, MCCB composite score and FC measures Pearson's r was used. Finally, mean differences between pre/post-VR symptoms scores were tested using paired t-test.

**Result:**

There was a significant difference between patients and controls on the verbal learning task (t16.38=−4.67,p < .001), and total time spent completing the VR task (t11.41 = 2.67, p = .023). In addition, VStore had a strong association with MCCB composite score (r=−.80,p = .010). While both VStore (r=−.82, p < 001) and MCCB (r = .77,p = .010) had significant correlation with the UPSA, only VStore had a significant association with the GAF (r=−.68,p = .030). Finally, VStore appears to be well-tolerated, causing no measurable side effects in the VRSQ (Pre-VR Mean =12.1[SD = 13.5], Post-VR Mean = 9.6[SD = 11.5],t9 = 0.49,p > .05).

**Conclusion:**

Results suggest that VStore can discriminate between schizophrenia patients and healthy controls. In addition, VStore and MCCB seem to be strongly associated, suggesting that they tap into identical cognitive domains. VStore seems to be strongly correlated with FC, more so than the MCCB, and cause no measurable side effects. Taken together, this suggests that this novel VR task has the potential to reliably measure cognition and FC simultaneously.

